# A Systematic Review and Meta‐Analysis of the Global Prevalence of Dry Mouth in Older Adults

**DOI:** 10.1111/ger.70076

**Published:** 2026-05-17

**Authors:** Porawit Kamnoedboon, Foteini Spyraki, W. Murray Thomson, Murali Srinivasan

**Affiliations:** ^1^ Clinic of General, Special Care, and Geriatric Dentistry, Center for Dental Medicine University of Zurich Zurich Switzerland; ^2^ Faculty of Dentistry Chulalongkorn University Bangkok Thailand; ^3^ Division of Gerodontology and Removable Prosthodontics, University Clinics of Dental Medicine University of Geneva Geneva Switzerland; ^4^ Emeritus Professor, Faculty of Dentistry University of Otago Dunedin New Zealand; ^5^ Honorary Professor, Center of Excellence in Genomics and Precision Dentistry, International Program in Geriatric Dentistry and Special Care, Faculty of Dentistry Chulalongkorn University Bangkok Thailand

**Keywords:** dry mouth, older adults, salivary gland hypofunction, xerostomia

## Abstract

**Objective:**

The objective of this study is to update prevalence estimates for xerostomia and salivary gland hypofunction (SGH) among older adults through a systematic review and meta‐analysis.

**Background:**

Dry mouth is common among older adults but is inconsistently assessed and reported. Xerostomia and SGH represent distinct conditions with different diagnostic approaches, leading to heterogeneity in prevalence estimates.

**Materials and Methods:**

A systematic literature search of Medline (PubMed) and Web of Science was conducted from January 2018 to March 2025. This was supplemented by manual backward citation tracking of the foundational review to identify all eligible pre‐2018 epidemiological studies. Only studies utilizing population‐representative samples of adults aged 50 years and older with defined diagnostic criteria were included. Risk of bias was formally assessed using the Joanna Briggs Institute (JBI) Critical Appraisal Tool for Prevalence Studies. Pooled prevalence estimates were calculated using random‐effects meta‐analyses. (PROSPERO: CRD420251009592).

**Results:**

A total of 37 studies—encompassing over 1.6 million participants—met the inclusion criteria. The pooled global prevalence of xerostomia among older adults was 21% (95% CI: 19%–23%). For SGH, the pooled prevalence measured via UWS was 12% (95% CI: 4%–24%), whereas SWS yielded a prevalence of 13% (95% CI: 3%–28%). High heterogeneity was observed across all analyses. Differences in diagnostic thresholds and saliva collection methods contributed to the observed variability.

**Conclusion:**

This updated meta‐analysis confirms that xerostomia affects around one‐fifth of older adults, while SGH is less common, depending on the diagnostic criteria used. Future research should focus on improving diagnostic consistency and sample representativeness to enable more consistent and robust estimates, and thereby better guide prevention and care in geriatric oral health.

## 1 Introduction

Among older adults, dry mouth is a common yet underdiagnosed condition [1]. It can manifest as either salivary gland hypofunction (SGH), an objectively determined low salivary flow rate, or xerostomia, the subjective sensation of oral dryness [2]. These entities, although commonly used interchangeably, are largely distinct clinical phenomena. Symptoms may be experienced by some in the absence of a measurably low flow rate secretion, and others with very low salivary flow rates may be asymptomatic. SGH was observed to be present in only 28.6% of those reporting xerostomia [3, 4]. This makes dry mouth diagnosis and prevalence estimation particularly challenging.

Depending on whether the focus is on the subjective or objective characteristics of dry mouth, different assessment methods are used. Xerostomia is typically evaluated by self‐report, using either a single global question (e.g., “How often does your mouth feel dry?”) or more detailed instruments such as the 11‐item Xerostomia Inventory (XI) [2], or its validated short‐form (SXI) [5], both of which aim to place respondents on a continuum of symptom severity. The single‐item approach is used to estimate prevalence. To measure SGH, salivary flow should be measured as either a stimulated salivary flow rate (SSFR) or an unstimulated salivary flow rate (USFR). Salivary flow is stimulated by gustatory or masticatory agents for SSFR, and the measured flow rates are categorized using cut‐off values. For USFR, the whole saliva is typically collected over a few minutes using various methods, such as spitting [6]. Discrepancies in diagnostic methods, particularly between objective salivary flow measurement and symptom assessment have been an important source of the heterogeneity in reported prevalence rates in older people. Other contributors to the observed variation in the reported prevalence of dry mouth in older people are heterogeneity in case definitions, diagnostic criteria, assessment methods, sampling, and the measurement of relevant exposure variables [7]. As a result, estimating the true burden of disease remains challenging.

Meta‐analyses of a condition's prevalence require the use of clear and unambiguous case definitions, along with data from representative samples (allowing generalization of findings back to the source population). Biased estimates can arise if either of those conditions has not been met; thus, a key step in identifying usable studies is the careful and detailed scrutiny of (a) how they measured the condition and (b) how they obtained their sample. For example, a meta‐analysis of SGH prevalence published five years ago (Pina et al., 2020) [8] reported a relatively high overall estimate of 33.4%, but five of the 13 studies included had used clinical convenience samples, and there was unacceptable variation in the flow rate criteria which had been used to define cases of SGH. Another meta‐analysis of dry mouth prevalence was conducted by Agostini et al. (2018), who examined the literature published on dry mouth up to February 2018 [1]. Some 5760 studies were screened, with 29 of those eventually included in their meta‐analysis, which gave an overall estimated dry mouth (whether SGH or xerostomia) prevalence of 22.6% (95% CI: 17.0 to 26.0) [1]. However, close scrutiny of the 28 papers involving xerostomia reveals that only eight had used appropriate measurement of the condition. Since that time, there have been 94 more papers reporting estimates for the prevalence of dry mouth. Thus, there is a need for more contemporary estimates for each of xerostomia and SGH, and for a more rigorous meta‐analysis which includes only studies which have used valid measurement of the aspect of dry mouth being investigated. Accordingly, the aim of this study was to update and improve the precision of the earlier work by conducting a meta‐analysis of the prevalence of dry mouth among older adults.

## 2 Materials and Methods

This systematic review and meta‐analysis was conducted in accordance with the Preferred Reporting Items for Systematic Reviews and Meta‐Analyses (PRISMA) 2020 guidelines [9, 10]. A protocol was prospectively registered in the International Prospective Register of Systematic Reviews (PROSPERO Registration ID: CRD420251009592), which specified the review objectives, inclusion criteria, data synthesis strategy, and risk of bias assessment.

### 2.1 Eligibility Criteria

Studies were considered eligible for inclusion if they met all of the following criteria:

1. Design: Original epidemiological investigations using observational designs, including cross‐sectional studies or cohort studies.
2. Population: General population‐based studies with representative samples, including adults aged ≥ 50 years. This age threshold (late middle age onwards) was selected as it represents the life stage where age‐associated chronic diseases and polypharmacy, primary drivers of dry mouth, typically begin to emerge [11, 12, 13]. Furthermore, participants were included irrespective of sex, setting, or comorbidity burden. This broad inclusion approach was chosen to ensure that the pooled prevalence estimates accurately reflect the whole general population, rather than a restricted, artificially healthy subset.
3. Condition and outcome measures: Studies reporting the prevalence of xerostomia and/or SGH using clearly defined diagnostic criteria. Xerostomia had to be assessed via self‐report (e.g., global question or validated instrument), while SGH measurement required objective salivary flow measurement.
4. Data Reporting: Articles had to report prevalence estimates directly or provide sufficient data from which prevalence could be calculated.

The exclusion criteria were equally specific. Studies were excluded if their sampling frame focused exclusively on clinical or convenience samples of patients with specific health conditions (e.g., patients with cancer, Sjögren's syndrome, asthma, or depression). To clarify, individuals with comorbidities were not excluded if they were part of a broader, representative general population sample; disease‐specific clinical studies were excluded because they do not provide true whole‐population epidemiological estimates. In vitro or animal experiments, or case–control studies, case series, reviews, commentaries, conference abstracts, or proof‐of‐concept studies, were also excluded. These exclusions were designed to ensure methodological rigour and generalizability of the findings (Table 1).

**TABLE 1:** Focus question, criteria for inclusion, sources of information, search terms, search strategy, search filters, and search dates.

Focus question	What is the global prevalence of dry mouth, including xerostomia and salivary gland hypofunction (SGH), among adults aged 50 years and older?	
Criteria	Inclusion criteria	Population‐based studies with representative samples Studies with clearly defined diagnostic criteria for xerostomia (self‐reported sensation of dry mouth) and/or SGH (objectively measured salivary flow). Original epidemiological investigations reporting the prevalence of xerostomia and/or SGH. Observational study designs, including cross‐sectional studies, cohort studies, and Studies with clearly reported the prevalence of xerostomia and/or hyposalivation or have included data allowing its calculation. Participants aged 50 years and older.
	Exclusion criteria	Studies focusing exclusively on clinical samples with specific health conditions (e.g., Sjögren's syndrome, asthma, cancer, depression) or convenience samples. In vitro, animal studies, and proof‐of‐concept experiments. Case–control studies, case reports, case series, reviews, conference abstracts, and commentaries.
Information sources	Electronic databases	MEDLINE (PubMed) and Web of Science.
	Journals	All peer reviewed journals available online in databases: Medline (PubMed) and Web of Science.
	Others	Popular online internet search engines (Google), Online internet research community websites (https://www.researchgate.net/), reference crosschecks, personal communications, hand‐searches.
	Grey literature	
	Language	Restriction to English
Search terms	Keywords included the following terms	Xerostomia, Dry Mouth, Mouth Dryness, Hyposalivation, Epidemiologic Studies, Cross‐Sectional Studies, Longitudinal Studies, Cohort Studies
Search query performed in the electronic databases, following the approach used by Agostini et al. (2018)	MEDLINE (PubMed) and Web of Science	((((“Xerostomia” OR “Dry Mouth”) OR “Mouth Dryness”) OR “Hyposalivation”) AND ((((((((((((((((((((((((“Epidemiological Studies” OR “Epidemiological Study”) OR “Cross Sectional Study”) OR “Cross Sectional Studies”) OR “Cross‐Sectional Study”) OR “Cross‐Sectional Studies”) OR “Studies, Cross‐Sectional”) OR “Study, Cross‐Sectional”) OR “Prevalence Studies”) OR “Prevalence Study”) OR “Studies, Prevalence”) OR “Study, Prevalence”) OR “Cohort Study”) OR “Cohort Studies”) OR “Incidence Study”) OR “Incidence Studies”) OR “Studies, Incidence”) OR “Study, Incidence”) OR “Follow up Studies”) OR “Follow‐up Studies”) OR “Follow up Study”) OR “Follow‐up Study”) OR “Prevalence”) OR “Incidence”)))
Filters	Year range	2018–2025 (This filter was applied to database searches only. Manual backward citation tracking of the foundational review captured eligible studies before 2018 without year restriction)
	Language	Not applied
	Species	Not applied
	Ages	Not applied
	Journal categories	Not applied
Search dates	January 2018 to Mar 2025	A final confirmatory online search was performed on 31 Mar 2025. No further online searches were conducted after this date.

### 2.2 Information Sources

The primary data sources for this review were the electronic databases Medline (via PubMed) and Web of Science, selected for their extensive coverage of peer‐reviewed health science literature. In addition to database searches, supplementary methods included grey literature searches using the popular online search engines (Google), professional research community websites (ResearchGate), hand‐searching reference lists of eligible studies and relevant reviews, and personal communications where applicable (Table 1). This was strictly supplemented by extensive manual backward citation searching of the previous foundational systematic review by Agostini et al. (2018) [1]. This cross‐checking strategy was used to capture all eligible pre‐2018 epidemiological studies, ensuring that the current review provides a definitive, chronologically complete population estimate.

### 2.3 Search Strategy

The search strategy was developed by adapting the approach used by Agostini et al. (2018) [1], ensuring sensitivity to the complex terminology used in the literature on dry mouth. The key terms used included: *xerostomia*, *dry mouth*, *mouth dryness*, *hyposalivation*, *epidemiologic studies*, *cross‐sectional studies*, *cohort studies*, *longitudinal studies*, *prevalence*, and *incidence*. These terms were incorporated into an extensive Boolean logic query applied to both Medline and Web of Science. No restrictions were placed on language, species, age group, or journal category. The publication date was restricted to the period between January 2018 and March 2025, ensuring inclusion of the most up‐to‐date research published since the previous review. A final confirmatory search was completed on 31 March 2025 (Table 1).

### 2.4 Selection Process

**FIGURE 1:**
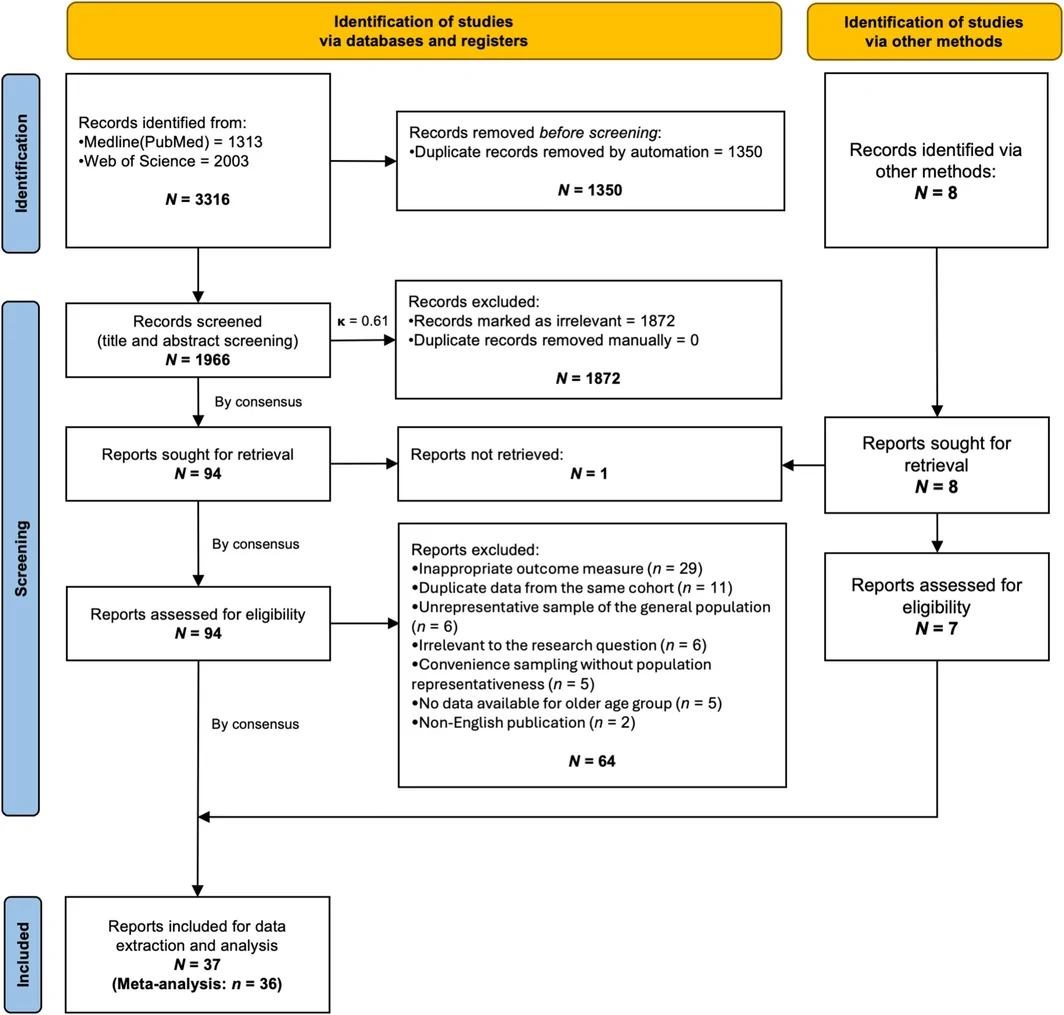
PRISMA flow diagram summarizing the study selection process, including records identified, duplicates removed, records screened, and studies included in the meta‐analysis. *N* or *n*, number; κ, Cohen's Kappa value. [Colour figure can be viewed at wileyonlinelibrary.com]

Following the automated removal of duplicate records using EndNote version 20.6, all retrieved articles were screened independently by two reviewers (PK and WMT) without the use of specialized screening software. First, titles and abstracts were reviewed to assess relevance based on the inclusion criteria. Second, full texts of potentially eligible studies were retrieved and assessed in detail. Any discrepancies between reviewers were resolved through consensus discussions involving all authors. Reasons for exclusion at the full‐text stage were documented. The entire selection process is illustrated in the PRISMA flow diagram (Figure 1).

### 2.5 Data Collection Process and Data Items

Data were extracted independently by the two reviewers using a pre‐structured extraction form, with any discrepancies resolved through discussion and consensus. The specific data items extracted for each included study comprised: general study characteristics (author, publication year, country, setting, and study design); sample characteristics (sample size, age range, and gender distribution); diagnostic methods for xerostomia or SGH (e.g., validated questionnaire, single‐item self‐report, or specific salivary flow measurement protocols); diagnostic thresholds and saliva collection methods, where applicable; and prevalence data (with 95% confidence intervals) or raw data permitting its calculation. If clarifications were required, the study authors were contacted directly.

### 2.6 Risk of Bias Assessment

The included studies' methodological quality and risk of bias were assessed using the Joanna Briggs Institute (JBI) Critical Appraisal Tool for Prevalence Studies [14]. Nine domains were assessed, including target population representation, sampling methods, sample size adequacy, detailed description of participants and setting, data analysis coverage, validity of condition identification, reliability of condition measurement, appropriateness of statistical analysis, and adequacy of the response rate. Disagreements were resolved through discussion and consensus. Based on the proportion of positive (“Yes”) responses, studies were categorized as having a low risk of bias (7–9 positive responses), moderate risk of bias (4–6 positive responses), or high risk of bias (0–3 positive responses).

### 2.7 Effect Measures

The primary outcome measure was the prevalence (%) of xerostomia and/or SGH in the study sample. Where necessary, we calculated prevalence estimates by dividing the number of affected individuals by the total sample size.

### 2.8 Synthesis Methods

Separate meta‐analyses were conducted for xerostomia and SGH. Owing to expected methodological and population heterogeneity, a random‐effects model using the DerSimonian and Laird method was applied [15, 16]. Before pooling, prevalence estimates were transformed using the Freeman‐Tukey double arcsine transformation to stabilize variances [17]. The meta‐analysis was conducted using MetaAnalysisOnline.com, an R‐based web application that incorporates the meta, metafor, and RTSA packages [18]. Heterogeneity among studies was quantified using the I^2^ statistic, with values above 75% indicating substantial heterogeneity [19].

### 2.9 Reporting Bias Assessment

To evaluate the potential for small‐study effects or publication bias, funnel plots were visually inspected and Egger's test was used for statistical assessment (*p* < 0.05) [20].

### 2.10 Certainty Assessment

A formal assessment of the certainty of evidence (e.g., using the GRADE framework) was not conducted for this review. Instead, the robustness of the prevalence estimates is considered in the Discussion, taking into account the methodological limitations and consistency of the included studies [21].

## 3 Results

### 3.1 Study Selection and Study Characteristics

A total of 3316 records were identified through electronic database searches, including 1313 from Medline (PubMed) and 2003 from Web of Science (Figure 1). After the removal of 1350 duplicate records via automation, 1966 unique records remained for title and abstract screening. Following independent screening by two reviewers (inter‐rater agreement κ = 0.61), 94 full‐text reports were assessed for eligibility. No additional studies were identified through other sources, and no articles were excluded due to retrieval issues. Of the 94 full‐text articles reviewed, 64 were excluded for the following reasons: inappropriate outcome measures (*n* = 29); duplicate data from the same sample (*n* = 11); unrepresentative population sample (*n* = 6); lack of relevance to the research question (*n* = 6); convenience sampling without population representativeness (*n* = 5); lack of data on older adults (*n* = 5); and non‐English publications (*n* = 2) (Supporting Information 1). An additional 7 eligible reports [4, 22, 23, 24, 25, 26, 27] were identified via manual reference cross‐checking of previous reviews. Ultimately, 37 studies [4, 12, 22, 23, 24, 25, 26, 27, 28, 29, 30, 31, 32, 33, 34, 35, 36, 37, 38, 39, 40, 41, 42, 43, 44, 45, 46, 47, 48, 49, 50, 51, 52, 53, 54, 55, 56] were included for data extraction and qualitative synthesis; of those, 36 studies [4, 12, 22, 23, 24, 25, 26, 27, 28, 29, 30, 31, 32, 33, 34, 35, 36, 37, 38, 39, 40, 41, 42, 43, 45, 46, 47, 48, 49, 50, 51, 52, 53, 54, 55, 56] were eligible for meta‐analysis.

### 3.2 Synthesis of Results: Descriptive Analysis of the Included Studies

A detailed summary of the included studies is presented in Table 2. A total of 37 studies [4, 12, 22, 23, 24, 25, 26, 27, 28, 29, 30, 31, 32, 33, 34, 35, 36, 37, 38, 39, 40, 41, 42, 43, 44, 45, 46, 47, 48, 49, 50, 51, 52, 53, 54, 55, 56] were included in the qualitative synthesis, encompassing diverse geographical regions, study designs, and assessment methods for xerostomia and SGH. Most included studies were cross‐sectional, while a smaller number were cohort studies. The addition of large‐scale national databases, such as the New Zealand national sample reported by Benn et al. (2015) [23] representing 1,483,560 participants, significantly increased the robustness of the pooled data, bringing the total sample size across all included studies to over 1.6 million participants. Study settings covered a wide range of countries, with Japan being the most represented in terms of the number of different studies. Studies used self‐report to assess xerostomia, using single‐item questions or validated tools such as the Xerostomia Inventory (XI) or the Summated Xerostomia Inventory (SXI). SGH was assessed in a subset of studies, using either unstimulated whole saliva (UWS) or stimulated whole saliva (SWS), with cut‐off thresholds of ≤ 0.1 mL/min for UWS and ≤ 0.7 mL/min for SWS. The reported prevalence of xerostomia varied widely, ranging from approximately 8% to over 50%, depending on population characteristics and assessment criteria. Fewer studies reported SGH prevalence, with observed rates differing by saliva collection methods and threshold definitions.

**TABLE 2:** Characteristics of included studies.

Author and year	Study design	Country	Participant age (years)	Female participants (%)	Xerostomia				SGH			
					Assessment & criteria	Cases	Total sample	Prevalence (%)	Assessment & criteria	Cases	Total sample	Prevalence (%)
Abbas et al. 2021^a^	Cohort	Japan	≥ 65	54.9	Self‐reported using JAGES questionnaire: “Do you often experience having a dry mouth?” (Yes/No)	4712	21867	21.5	—	—	—	—
Abe et al. 2024^a^	Cohort	Japan	≥ 75	57.3	Self‐reported questionnaire: considered as having dry mouth if participants answered “yes” to ≥ 2 of the following: “mouth feels dry”, “tongue hurts”, “taste has decreased”; or 1 positive response plus one of: ≥ 5 oral meds/day, poor nutrition, or dentist‐suspected dryness based on visual inspection/palpation	4436	22747	19.5	—	—	—	—
Albani et al. 2021^a^	Cross‐sectional	UK	≥ 85	62	Self‐reported: presence of any symptoms (e.g., sipping water at night, feeling dry mouth when eating)	462	853	54.2	Salivary volume < 0.83g/3min (lowest tertile)	—	—	—
Anttila et al. 1998^a^	Cross‐sectional	Finland	55	55.8	Interview question with responses: never/rarely, sometimes, often. Xerostomia defined as “sometimes” or “often” (combined as sensation of dry mouth)	1944	214	11.0	For UWS, hyposalivation defined as ≤ 0.1 mL/min	—	—	—
									For SWS, hyposalivation defined as ≤ 0.7 mL/min	—	—	—
Astrom et al. 2019^a^	Cohort	Sweden & Norway	65‐70	Sweden: 51.2; Norway: 48.8%	Self‐reported via two questions: “Do you feel dry mouth during daytime?” and “Do you feel dry mouth during night‐time?” with responses (1) yes often, (2) yes sometimes, (3) no seldom, (4) no never. Dichotomized into xerostomia (yes often/sometimes) vs. no (seldom/never)	2190	7836	27.9	—	—	—	—
Bakker et al. 2020^a^	Cross‐sectional	Netherland	≥ 75	55.3	Self‐reported dry mouth in the last 3 months via questionnaire	190	1622	11.7	—	—	—	—
Benn et al. 2015^a^	Cross‐sectional	New Zealand	≥ 60	52.6	Single interview item: “How often does your mouth feel dry?” (Never/Occasionally/Frequently/Always)	218083	1483560	14.7	—	—	—	—
Castrejon‐Perez et al. 2023^a^	Cross‐sectional	Mexico	≥ 70	52.1	Self‐reported: “Do you frequently experience mouth dryness?” (Yes/No)	212	487	43.5	—	—	—	—
Crews et al. 2020^a^	Cross‐sectional	United States	≥ 65	57.5	Self‐reported dry mouth in the past 6 months (part of a composite question on mouth problems in the NHIS Oral Health Module)	628	3863	16.3	—	—	—	—
da Silva et al. 2017^a^	Cohort	Brazil	50‐59	55.5	How often do you experience dry mouth? (Never/Sometimes/Frequently/Always). Xerostomia defined as “Frequently” or “Always”	68	321	21.2	—	—	—	—
Diep et al. 2021^a^	Cross‐sectional	Norway	65	48	Single standardized question: “How often does your mouth feel dry?” (Responses: Never, Occasionally, Frequently, Always). Participants answering “Frequently” or “Always” were classified as having xerostomia; Additionally assessed with the Summated Xerostomia Inventory–Dutch Version (SXI‐D)	45	460	9.8	For UWS, hyposalivation defined as ≤ 0.1 mL/min	UWS: 36	UWS: 457	7.9
									For SWS, hyposalivation defined as ≤ 0.7 mL/min	SWS: 18	SWS: 457	3.9
Drachev et al. 2022^a^	Cross‐sectional	Lithuania	65‐74	69	Self‐reported; participants were asked if they experienced dry mouth (response options: never/sometimes/often/always). Those reporting “sometimes”, “often” or “always” were considered as having xerostomia	109	370	29.5	—	—	—	—
Fornari et al. 2021^a^	Cross‐sectional	Brazil	≥ 60	44	Self‐reported xerostomia via Xerostomia Inventory (XI); classified based on the single item “My mouth feels dry” with response options “fairly often” or “very often” considered positive	56	293	19.1	—	—	—	—
Hynne et al. 2022^a^	Cross‐sectional	Norway	65	55	General xerostomia question: “How often does your mouth feel dry?” (response ≥ 3 = xerostomia case); Summated Xerostomia Inventory‐Dutch version (SXI), score > 10 used as case definition	12	150	8	For UWS, hyposalivation defined as ≤ 0.1 mL/min	UWS: 12	UWS: 150	8
									For SWS, hyposalivation defined as ≤ 0.7 mL/min	SWS: 7	SWS: 148	4.7
Ianunzio et al. 2019^a^	Cross‐sectional	Brazil	50‐59	56	Self‐reported: “How often does your mouth feel dry?”; responses “often” and “always” were categorized as xerostomia	44	349	12.6	—	—	—	—
Iwasaki et al. 2018^a^	Cohort	Japan	70	49	Self reported: “Does your mouth feel dry?” (Yes/No)	—	—	—	SWS, hyposalivation defined as < 0.7 mL/min	SWS: 85	SWS: 306	27.8
Iwasaki et al. 2023^a^	Cross‐sectional	Japan	≥ 65	62	Single‐item question from the Kihon Checklist: “Do you often experience having a dry mouth?”	153	607	25.2	—	—	—	—
Iwasaki et al. 2024^a^	Cross‐sectional	Japan	≥ 65	52	Self reported: “Do you often experience having a dry mouth?” (Yes/No); from the Oral Frailty 5‐Item Checklist (OF‐5)	321	1206	26.6	—	—	—	—
Jamieson et al. 2020^a^	Cross‐sectional	Australia	≥ 55	51	Self reported: “How often does your mouth feel dry?”; participants answering “frequently” or “always” were classified as having xerostomia	1108	5683	19.5	—	—	—	—
Johansson et al. 2012^a^	Cross‐sectional	Sweden	75	53.2	Two separate questions: (1) “Does your mouth usually feel dry in the daytime?”, (2) “Does your mouth usually feel dry at night?”. Response: yes often/yes sometimes/no seldom/no never. Xerostomia defined as: yes often/yes sometimes	1325	3539	37.4	—	—	—	—
Johansson et al. 2020^a^	Cross‐sectional	Sweden	70	52	Two self‐reported questions from a questionnaire: (a) “Does your mouth usually feel dry at night?” (b) “Does your mouth usually feel dry in the daytime?” Response options: “yes, often”; “yes, sometimes”; “no, seldom”; “no, never”; Participants answering “yes, often” or “yes, sometimes” considered as having xerostomia	1071	3585	29.9	—	—	—	—
Johansson et al. 2022	Cross‐sectional	Sweden	75	—	Self‐reported question: “Do you have dry mouth during daytime/night‐time?” with response options “Yes, often/sometimes” vs. “No, rarely/never”	—	—	—	—	—	—	—
Karawekpanyawong et al. 2022^a^	Cross‐sectional	Japan	90	56	—	—	—	—	SWS, hyposalivation defined as ≤ 0.7 mL/min	SWS: 18	SWS: 84	21.4
Kubo et al. 2024^a^	Cohort	Japan	65‐80	48.6	Single‐item self‐report from the Kihon Checklist: “Do you often experience having a dry mouth?” (Yes/No)	685	3594	19.1	—	—	—	—
Lin et al. 2022^a^	Cross‐sectional	Taiwan	≥ 65	71.4	Condensed version of the Xerostomia Inventory with 5 items; each rated from 1 (never) to 3 (often). Scores ≥ 10 (top 50%) indicated xerostomia	128	1100	11.6	—	—	—	—
			65‐74	69.5		50	639	7.8	—	—	—	—
			≥ 75	74.2		78	461	16.9	—	—	—	—
Lu et al. 2020^a^	Cross‐sectional	Taiwan	74	71.4	Condensed version of the Xerostomia Inventory (5 items scored 1–3, total score 5–15); score ≥ 10 indicated xerostomia	119	1076	11.1	—	—	—	—
Morishita et al. 2021^a^	Cohort	Japan	≥ 65	76.8	Oral dryness was assessed clinically using a four‐point scale based on the classification by Kakinoki classification. Presence was defined as a score of 1 or higher (i.e., saliva viscosity or drier)	57	289	19.7	—	—	—	—
Ohara et al. 2022^a^	Cohort	Japan	66‐77	59.8	Self‐reported: “Does your mouth feel dry?” (Yes/No); from the Kihon Checklist	166	609	27.3	—	—	—	—
Pérez‐González et al. 2021^a^	Cross‐sectional	Spain	≥ 50	64.7	Assessed using the validated Spanish version of the Xerostomia Inventory (XI) (0–11: No; 12–22: Mild; 23–33: Moderate; 34–44: Severe)	117	353	33.1	—	—	—	—
Quandt et al. 2011^a^	Cross‐sectional	United States	≥ 60	54.4	11‐item Xerostomia Inventory (XI). Also included single question: “How often does your mouth feel dry?” (never/occasionally/frequently/always). Xerostomia prevalence defined from the single question as “frequently” or “always”	158	622	25.4	—	—	—	—
Shiota et al. 2023^a^	Cohort	Japan	≥ 65	52	Self‐reported, from the Kihon checklist: “Do you often experience having a dry mouth?” (Yes/No)	11860	63602	18.6	—	—	—	—
So et al. 2010^a^	Cross‐sectional	South Korea	52‐63	57.0	9‐item dry mouth questionnaire (symptoms +behaviours; multiple‐item instrument)	261	1012	25.8	—	—	—	—
Stankeviciene et al. 2021^a^	Cross‐sectional	Lithuania	≥ 60	66.8	Self‐reported: “How often does your mouth feel dry?”; responses ‘often’ or ‘always’ were categorized as xerostomia	67	513	13.1	—	—	—	—
Tanaka et al. 2023^a^	Cohort	Japan	≥ 65	50.4	Self‐reported dry mouth using the Kihon Checklist question: “Do you often experience having a dry mouth?” (Yes/No)	563	2031	27.7	—	—	—	—
Thomson et al.1999^a^	Cross‐sectional	Australia	≥ 65	53.2	Single CATI question: “How often does your mouth feel dry?”. Response options: Never/Occasionally/Frequently/Always. Xerostomia defined as “Frequently” or “Always”	140	684	20.5	UWS, hyposalivation defined as < 0.1 mL/min	151	684	22.1
Thomson et al. 2021^a^	Cross‐sectional	New Zealand	≥ 65	67.5	Self‐reported: “How often does your mouth feel dry?”; responses “Always” or “Frequently” were classified as xerostomia	453	1525	29.7	—	—	—	—
Wang et al. 2020^a^	Cohort	New Zealand	≥ 60	46	Self‐reported via the validated 5‐item Summated Xerostomia Inventory–Dutch Version (SXI‐D); Also classified using a single question: “How often does your mouth feel dry?”, with “Frequently” or “Always” indicating xerostomia	130	627	20.7	—	—	—	—

*Note:* —, data not reported in the article.

*Abbreviations:* SGH, salivary gland hypofunction; SWS, stimulated whole saliva; UWS, unstimulated whole saliva.

^a^ Included in the meta‐analysis.

### 3.3 Synthesis of Results: Meta‐Analysis

A total of 36 studies [4, 12, 22, 23, 24, 25, 26, 27, 28, 29, 30, 31, 32, 33, 34, 35, 36, 37, 38, 39, 40, 41, 42, 43, 45, 46, 47, 48, 49, 50, 51, 52, 53, 54, 55, 56] were eligible for quantitative synthesis. Among these, 34 studies [4, 12, 22, 23, 24, 25, 26, 27, 28, 29, 30, 31, 32, 33, 34, 35, 36, 37, 38, 39, 41, 42, 43, 46, 47, 48, 49, 50, 51, 52, 53, 54, 55, 56] involving 1,640,079 participants contributed to the pooled prevalence estimate for xerostomia. Additionally, 3 studies [4, 36, 38] involving 1291 participants provided data on SGH using UWS, and 4 studies [36, 38, 40, 45] involving 995 participants reported SGH prevalence based on SWS.

**FIGURE 2:**
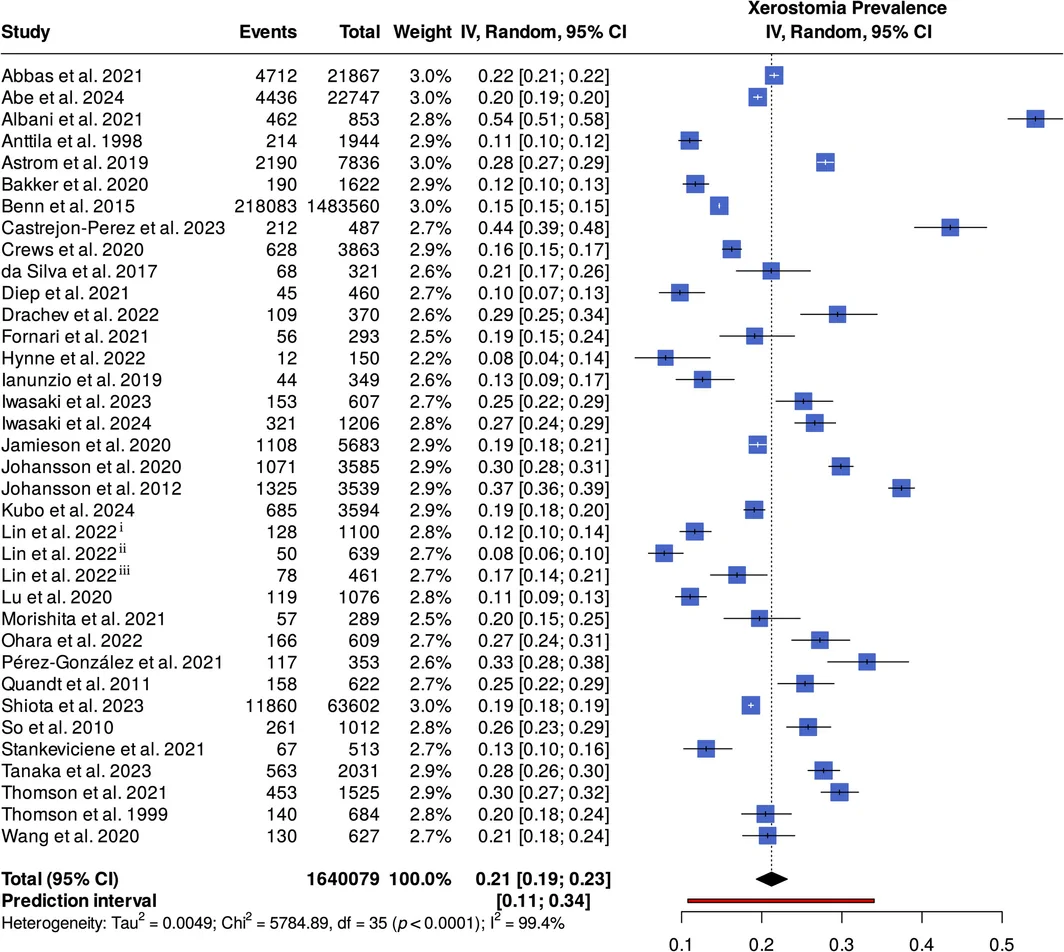
Forest plot showing the pooled prevalence of xerostomia across 34 studies involving adults aged ≥ 50 years (*n* = 1,640,079). The meta‐analysis was conducted using a random‐effects model with Freeman‐Tukey double arcsine transformation. [Colour figure can be viewed at wileyonlinelibrary.com]

The pooled prevalence of xerostomia across 34 studies was 21% (95% CI: 19 to 23%) (Figure 2). A random‐effects model with Freeman‐Tukey double arcsine transformation was used. Considerable heterogeneity was observed (I^2^ = 99.4%, *p* < 0.0001). The prediction interval ranged from 11% to 34%.

**FIGURE 3:**
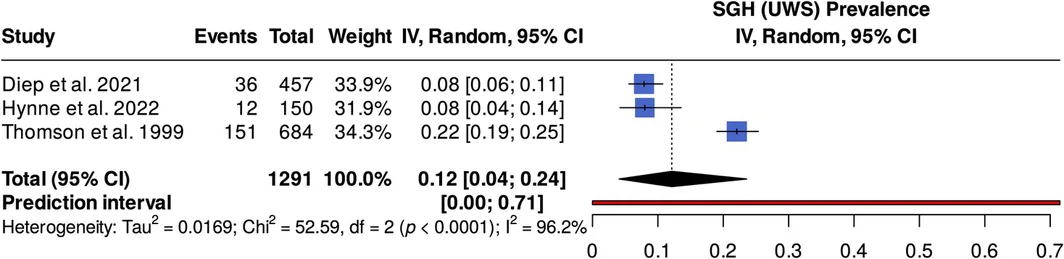
Forest plot showing the pooled prevalence of salivary gland hypofunction (SGH) based on unstimulated whole saliva (UWS) measurements (≤ 0.1 mL/min), derived from 3 studies (*n* = 1291). [Colour figure can be viewed at wileyonlinelibrary.com]

**FIGURE 4:**
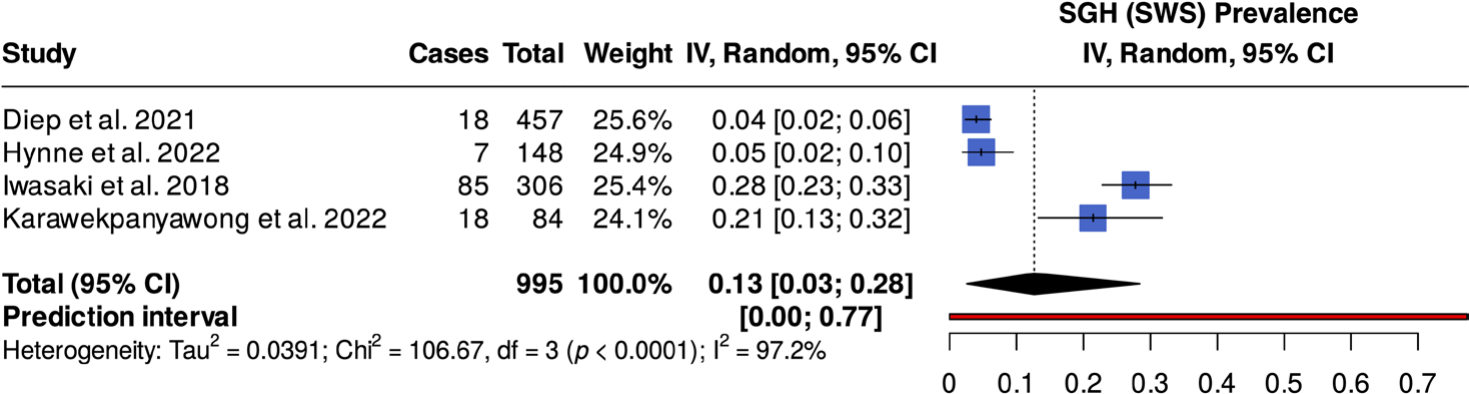
Forest plot showing the pooled prevalence of salivary gland hypofunction (SGH) based on stimulated whole saliva (SWS) measurements (≤ 0.7 mL/min), based on 4 studies (*n* = 995). [Colour figure can be viewed at wileyonlinelibrary.com]

Three studies assessed SGH via UWS, applying a diagnostic threshold of ≤ 0.1 mL/min (Figure 3). The pooled prevalence was 12% (95% CI: 4 to 24%) with high heterogeneity (I^2^ = 96.2%, *p* < 0.0001). The prediction interval ranged from 0% to 71%. Four studies evaluated SGH via SWS, with a threshold of ≤ 0.7 mL/min (Figure 4). The pooled prevalence was 13% (95% CI: 3 to 28%) with substantial heterogeneity (I^2^ = 97.2%, *p* < 0.0001). The wide prediction interval (0% to 77%) indicates a high degree of between‐study variability.

Sensitivity analyses were conducted to assess the impact of high‐prevalence studies on the overall xerostomia estimate. Excluding Albani et al. (2021) [30] resulted in a pooled prevalence of 20% (95% CI: 19 to 22%; I^2^ = 99.3%), while excluding Castrejon‐Perez et al. (2023) [33] yielded a prevalence of 21% (95% CI: 19 to 23%; I^2^ = 99.4%). When both studies were excluded, the pooled estimate remained at 20% (95% CI: 18 to 22%; I^2^ = 99.3%). These findings indicate that the overall estimate was robust and not substantially influenced by individual higher‐prevalence studies. Detailed results are provided in the Supporting Information 2.

### 3.4 Publication Bias

Visual inspection of the funnel plot for xerostomia prevalence indicated potential publication bias, which was statistically supported by Egger's test revealing significant funnel plot asymmetry (intercept: 7.92, 95% CI: 4.18 to 11.66; t = 4.147, *p* < 0.0001). Conversely, no significant evidence of publication bias or funnel plot asymmetry was detected for the SGH meta‐analyses, neither for UWS (intercept: 9.44, 95% CI: −32.48 to 13.59; t = −0.804, *p* = 0.569) nor for SWS (intercept: 4.89, 95% CI: −18.12 to 27.9; t = 0.416, *p* = 0.717). Detailed funnel plots are provided in the Supporting Information 3.

### 3.5 Risk of Bias Assessment

The complete risk‐of‐bias assessment for all 37 included studies is presented in Supporting Information 4. Overall, the methodological quality of the included reports was strong, presenting moderate to low risk of bias. The domains with the highest compliance were the adequacy of the sample size (Q3) and the detailed description of study participants and settings (Q4). Conversely, the most common methodological shortcomings across the literature were unclear or inadequate response rates (Q9) and sampling methods that did not optimally represent the target population (Q2).

## 4 Discussion

This meta‐analysis sought to provide an updated and methodologically rigorous synthesis of the global prevalence of dry mouth among older adults. To do so, we reviewed studies that used clearly defined diagnostic criteria and recruited representative population samples. Three separate meta‐analyses were performed to estimate the prevalence of xerostomia, SGH based on UWS and SWS. The pooled prevalence estimate for xerostomia was 21% (95% CI: 19 to 23%), based on 34 studies and over 1.6 million participants. The estimated prevalence for SGH using the UWS criterion (≤ 0.1 mL/min) was 12% (95% CI: 4 to 24%), based on three studies. Using the SWS criterion (≤ 0.7 mL/min), the SGH prevalence was estimated at 13% (95% CI: 3 to 28%), derived from four studies. Notably, substantial heterogeneity was present in all analyses.

A major strength of this review is its comprehensive chronological scope. By strategically integrating newly published data with foundational pre‐2018 epidemiological estimates, this review provides a highly robust population‐level estimate. By applying rigorous inclusion criteria, we restricted the analysis to studies that used validated definitions and population‐representative samples, thereby enhancing the validity and generalizability of our findings. Unlike previous syntheses, which have included convenience samples or poorly defined dry mouth measurements, this review provides clearer prevalence estimates that can help to inform the development of clinical and public health priorities. Moreover, the separate analyses for xerostomia and the two forms of SGH underscore the conceptual and clinical distinction between symptom perception and actual salivary function.

There are limitations to this review that should be acknowledged. First, the number of studies that met all inclusion criteria for SGH (particularly for UWS‐based SGH) was limited, and this may affect the statistical precision and generalizability of these estimates. Furthermore, this low number of well‐conducted studies restricted our ability to perform meaningful subgroup analyses, such as by specific age groups, functional status, or geographical region, because the low N within these strata would severely compromise the statistical validity of any such estimates. Second, although we made efforts to include only studies with validated and clearly reported measurement tools, the overall heterogeneity in the xerostomia and SWS data underscores the ongoing inconsistencies in the measurement of dry mouth, even in the recent literature. This is particularly evident for SGH, where the logistical challenges of collecting saliva in field surveys lead to substantial variation in protocols and adherence. It is also possible that some of the heterogeneity may have arisen from differences in participant characteristics, along with cultural differences in symptom reporting. Ideally, this heterogeneity would have been examined quantitatively and the associated risk factors synthesized; however, the profound variation across studies in how these variables were measured and analysed precluded such statistical exploration.

In comparing our findings with previous reviews, our estimated xerostomia prevalence (21%) is similar to the 22.6% reported by Agostini et al. in their 2018 meta‐analysis [1]. However, our findings are likely more accurate due to our inclusion of only methodologically sound studies, which deliberately excluded unrepresentative convenience samples and focused solely on methodologically sound, population‐based studies. By contrast, the previous estimate of SGH prevalence reported by Pina et al. (2020) was markedly higher, at 33.4%, likely due to the inclusion of clinical convenience samples and inconsistent case definitions [8]. Our lower estimates for SGH (8% for UWS, 13% for SWS) likely reflect more stringent inclusion criteria and standardized diagnostic thresholds. These differences highlight the importance of harmonizing measurement protocols and ensuring representativeness in prevalence studies of dry mouth.

These updated prevalence estimates have important implications for clinical risk assessment and population‐level surveillance. Xerostomia and SGH are now recognized as major contributors to poor oral health–related quality of life (OHRQoL) among older people, alongside tooth loss and untreated dental caries [57]. From a clinical perspective, dry mouth, especially when self‐reported, should serve as a trigger for a broader diagnostic and management strategy. This should include comprehensive oral health assessment, review of systemic health, and critically a medication review [13]. The latter is particularly salient given the high prevalence of polypharmacy in older adults and the known xerogenic potential of several commonly prescribed medications. Emerging evidence suggests that structured medication review can reduce unnecessary drug burden and potentially mitigate symptoms of dry mouth [13].

A particularly important methodological implication arising from our findings is the persistent inconsistency in how xerostomia is measured. Two decades after the call for improved epidemiological approaches by Thomson (2005) [7], many studies continue to use unvalidated or poorly worded questions to capture xerostomia. The single‐item self‐report remains the most commonly used tool, but the wording of such items varies considerably across studies. Subtle differences in phrasing can significantly influence prevalence estimates, underscoring the urgent need for consensus on a standardized, validated approach to xerostomia measurement. Only then can we ensure comparability across studies and obtain valid population‐level estimates.

The present findings affirm the continued relevance of dry mouth as an important health concern in ageing populations, with direct implications for oral and systemic health. The marked discrepancy between subjective and objective measures reinforces the need for both domains to be routinely evaluated in clinical settings. Accordingly, it is advocated that there is:

1. The urgent adoption of standardized definitions and validated instruments in dry mouth research.
2. The mandatory integration of dry mouth screening in geriatric care protocols.
3. The implementation of interprofessional approaches to dry mouth management, including routine medication review.

Addressing these areas will be critical in reducing the burden of dry mouth and improving quality of life for older adults.

## 5 Conclusion

This meta‐analysis confirms that xerostomia affects around one in five older adults, while SGH is less prevalent, with estimates depending on the diagnostic criteria used. Future research should focus on improving diagnostic consistency and representativeness to better guide prevention and care in geriatric oral health.

## Author Contributions

**Porawit Kamnoedboon:** conceptualization, methodology, validation, formal analysis, investigation, data curation, writing – original draft, writing – review and editing, visualization. **Foteini Spyraki:** conceptualization, methodology, validation, investigation, data curation, writing – original draft, writing – review and editing. **W. Murray Thomson:** conceptualization, methodology, validation, investigation, data curation, writing – original draft, writing – review and editing, supervision. **Murali Srinivasan:** conceptualization, methodology, validation, investigation, resources, data curation, writing – original draft, writing – review and editing, supervision.

## Funding

The authors have nothing to report.

## Ethics Statement

This study was based on previously published data and did not involve the collection of new human data. Therefore, ethical approval and informed consent were not required.

## Conflicts of Interest

The authors declare no conflicts of interest.

## Supporting information


**Supporting Information: 1** Excluded articles with reasons.

**Supporting Information: 2** Sensitivity analyses for the meta‐analysis of xerostomia prevalence.

**Supporting Information: 3** Publication biases.

**Supporting Information: 4** Risk of Bias Assessment of Included Studies, using the JBI Critical Appraisal Tool for Prevalence Studies.

## Data Availability

The data that support the findings of this study are available from the corresponding author upon reasonable request.
