# Supplementary material for: A Systematic Review and Meta‐Analysis of the Global Prevalence of Dry Mouth in Older Adults
Source: Gerodontology. 2026 May 17;43(3):383–402. doi: 10.1111/ger.70076 (PMC13498937; doi:10.1111/ger.70076)
Supplement: Supplementary file 1 [file GER-43-383-s001.docx]

**Supplementary Material 3.** Publication biases

**1. Funnel plot of included studies in the meta-analysis of xerostomia prevalence**

The funnel plot indicates a potential publication bias. The Egger's test supports the presence of funnel plot asymmetry (intercept: 7.92, 95% CI:4.18 - 11.66, t: 4.147, *p*-value < 0.0001).

**2. Funnel plot of included studies in the meta-analysis of salivary gland hypofunction (SGH) prevalence via unstimulated whole saliva (UWS)**

The funnel plot does not indicate a potential publication bias. The Egger's test does not support the presence of funnel plot asymmetry (intercept: -9.44, 95% CI:-32.48 - 13.59, t: -0.804, *p*-value: 0.569).

**3. Funnel plot of included studies in the meta-analysis of salivary gland hypofunction (SGH) prevalence via stimulated whole saliva (SWS)**

The funnel plot shows no apparent indication of publication bias. Additionally, Egger’s test revealed no significant evidence of funnel plot asymmetry (intercept = 4.89, 95% CI: –18.12 to 27.9; t = 0.416; *p* = 0.717).
