# Supplementary material for: A Systematic Review and Meta‐Analysis of the Global Prevalence of Dry Mouth in Older Adults
Source: Gerodontology. 2026 May 17;43(3):383–402. doi: 10.1111/ger.70076 (PMC13498937; doi:10.1111/ger.70076)
Supplement: Supplementary file 2 [file GER-43-383-s002.docx]

**Supplementary Material 2.** Sensitivity analyses for the meta-analysis of xerostomia prevalence

**1. Sensitivity analysis conducted after removing Albani et al. (2021)^1^**

**2. Sensitivity analysis conducted after removing Castrejon-Perez et al. (2023)^2^**

**3. Sensitivity analysis conducted after removing Albani et al. (2021)^1^ and Castrejon-Perez et al. (2023)^2^**

1.	Albani V, Nishio K, Ito T, et al. Associations of poor oral health with frailty and physical functioning in the oldest old: results from two studies in England and Japan. BMC GERIATRICS*.* 2021;21(1).

2.	Castrejon-Perez RC, Wanyonyi KL, Garcia-Vazquez PE, Cruz-Hervert LP, Ramirez-Aldana R, Borges-Yanez SA. Frailty index and ten oral conditions in the Coyoacan cohort study: A cross-sectional analysis. GERODONTOLOGY*.* 2023;40(3):372-381.
