# Supplementary material for: A Systematic Review and Meta‐Analysis of the Global Prevalence of Dry Mouth in Older Adults
Source: Gerodontology. 2026 May 17;43(3):383–402. doi: 10.1111/ger.70076 (PMC13498937; doi:10.1111/ger.70076)
Supplement: Supplementary file 3 [file GER-43-383-s003.docx]

**Supplementary Material 1.** Excluded articles with reasons

| **Author and Year** | **Exclusion Reason** |
| --- | --- |
| Adnam et al. 2020 | Not a representative sample of general population |
| Adolfsson et al. 2022 | Not a representative sample of general population |
| Ahonen et al. 2022 | No old age group data available |
| Alharbi et al. 2020 | Inappropriate measure |
| Alwaheidi et al. 2021 | Inappropriate measure |
| Angeles-Zurita et al. 2022 | Inappropriate measure |
| Beker et al. 2019 | Inappropriate measure |
| Botelho et al. 2020 | Inappropriate measure |
| Branco et al. 2024 | Convenience sample |
| Buhl et al. 2022 | Convenience sample |
| Cassiano (Schertel-Cassiano) et al. 2024 | Inappropriate measure |
| Chen et al. 2023 | Not a representative sample of general population |
| Choi et al. 2021 | Convenience sample |
| de Carvalho et al. 2024 | Not a representative sample of general population |
| Doi et al. 2023 | Inappropriate measure |
| Ganbavale et al. 2025 | Inappropriate measure |
| Ganbavale et al. 2024 | Same cohort |
| Goergen et al. 2023 | No old age group data available |
| Henricsson et al. 2024 | Inappropriate measure |
| Horncastle et al. 2024 | Same cohort |
| Ishimura et al. 2024 | Inappropriate measure |
| Islas-Granillo et al. 2019 | Convenience sample |
| Kakoei et al. 2021 | Inappropriate measure |
| Kawamoto et al. 2021 | Inappropriate measure |
| Khoury et al. 2022 | Inappropriate measure |
| Kiesswetter et al. 2020 | No old age group data available |
| Kim et al. 2021 | Not in English |
| Kotronia et al. 2021c | Same cohort |
| Kotronia et al. 2021c | Same cohort |
| Kotronia et al. 2019 | Same cohort |
| Kotronia et al. 2021 | Same cohort |
| Kotronia et al. 2021a | Same cohort |
| Kotronia et al. 2021b | Same cohort |
| Kudo et al. 2024 | Not relevant |
| Kunrath et al. 2021 | Same cohort |
| Kunrath et al. 2021 | Same cohort |
| Kuze et al. 2022 | Inappropriate measure |
| Langari et al. 2022 | Not a representative sample of general population |
| Laugisch et al. 2021 | Not a representative sample of general population |
| Lindroos et al. 2018 | Inappropriate measure |
| Ma et al. 2024 | Inappropriate measure |
| Mao et al. 2019 | Inappropriate measure |
| Mascarenhas et al. 2025 | Inappropriate measure |
| Mello et al. 2021 | Inappropriate measure |
| Morita et al. 2023 | Inappropriate measure |
| Nekouie et al. 2022 | Not relevant |
| Ohara et al. 2022a | Not relevant |
| Omidpanah et al. 2021 | Inappropriate measure |
| Oura et al. 2021 | Not relevant |
| Pérez‑Jaród et al. 2022 | Inappropriate measure |
| Puranen et al. 2023 | Inappropriate measure |
| Ramsay et al. 2018 | Convenience sample |
| Rech et al. 2019 | Inappropriate measure |
| Ribeiro Silva et al. 2019 | Not in English |
| Rodakowska et al. 2022 | No old age group data available |
| Saarela et al. 2022 | Not relevant |
| Saarela et al. 2019 | Same cohort |
| Salmi et al. 2022 | Inappropriate measure |
| Schoebrechts et al. 2024 | Inappropriate measure |
| Shimazaki et al. 2020 | Inappropriate measure |
| Stankeviciene et al. 2021 | Same cohort |
| Thomson et al. 2019 | No old age group data available |
| Torres et al. 2019 | Not relevant |
| Wu et al. 2024 | Inappropriate measure |
| Yamamoto-Kuramoto  et al. 2023 | Same cohort |
| Yu et al. 2021 | Inappropriate measure |
