# Supplementary material for: A Systematic Review and Meta‐Analysis of the Global Prevalence of Dry Mouth in Older Adults
Source: Gerodontology. 2026 May 17;43(3):383–402. doi: 10.1111/ger.70076 (PMC13498937; doi:10.1111/ger.70076)
Supplement: Supplementary file 4 [file GER-43-383-s004.docx]

**Supplementary Material 4:** Risk of Bias Assessment of Included Studies, using the JBI Critical Appraisal Tool for Prevalence Studies

| **Study (Author, Year)** | **Q1** | **Q2** | **Q3** | **Q4** | **Q5** | **Q6** | **Q7** | **Q8** | **Q9** | **Overall Score (Y/9)** | **Risk of Bias** |
| --- | --- | --- | --- | --- | --- | --- | --- | --- | --- | --- | --- |
| Abbas et al., 2021 | N | Y | Y | Y | N | Y | Y | Y | N | 6/9 | Moderate |
| Abe et al., 2024 | N | N | Y | Y | Y | Y | U | Y | U | 5/9 | Moderate |
| Albani et al., 2021 | Y | Y | Y | Y | U | Y | Y | Y | U | 7/9 | Low |
| Anttila et al., 1998 | Y | Y | Y | Y | U | U | Y | Y | Y | 7/9 | Low |
| Astrøm et al., 2019 | Y | Y | Y | Y | Y | U | Y | Y | Y | 8/9 | Low |
| Bakker et al., 2020 | U | Y | U | Y | N | U | U | Y | N | 3/9 | High |
| Benn et al., 2015 | Y | Y | Y | Y | Y | Y | Y | Y | U | 8/9 | Low |
| Castrejón-Pérez et al., 2023 | Y | N | U | Y | N | Y | U | Y | N | 4/9 | Moderate |
| Crews et al., 2020 | Y | Y | Y | Y | Y | Y | Y | Y | U | 8/9 | Low |
| da Silva et al., 2017 | Y | Y | Y | Y | U | Y | Y | Y | U | 7/9 | Low |
| Diep et al., 2021 | Y | Y | Y | Y | Y | Y | Y | Y | U | 8/9 | Low |
| Drachev et al., 2022 | Y | Y | U | Y | Y | Y | Y | Y | N | 7/9 | Low |
| Fornari et al., 2021 | Y | Y | Y | Y | Y | Y | Y | Y | Y | 9/9 | Low |
| Hynne et al., 2022 | Y | N | U | Y | Y | Y | Y | Y | N | 6/9 | Moderate |
| Ianunzio et al., 2019 | Y | Y | Y | Y | Y | Y | Y | Y | Y | 9/9 | Low |
| Iwasaki et al., 2018 | Y | U | Y | Y | Y | Y | Y | Y | U | 7/9 | Low |
| Iwasaki et al., 2023 | Y | N | Y | Y | Y | Y | Y | Y | N | 7/9 | Low |
| Iwasaki et al., 2024 | Y | U | Y | Y | Y | Y | Y | Y | U | 7/9 | Low |
| Jamieson & Thomson, 2020 | Y | U | Y | Y | U | Y | Y | Y | U | 6/9 | Moderate |
| Johansson et al., 2012 | Y | Y | Y | Y | Y | U | Y | Y | Y | 8/9 | Low |
| Johansson et al., 2020 | Y | Y | Y | Y | U | U | Y | Y | U | 6/9 | Moderate |
| Johansson et al., 2022 | Y | Y | Y | Y | Y | U | Y | Y | Y | 8/9 | Low |
| Karawekpanyawong et al., 2023 | N | N | N | Y | Y | Y | U | Y | U | 4/9 | Moderate |
| Kubo et al., 2024 | Y | N | Y | Y | N | Y | U | Y | U | 5/9 | Moderate |
| Lin et al., 2022 | N | U | Y | Y | Y | Y | Y | Y | Y | 7/9 | Low |
| Lu et al., 2020 | N | U | Y | Y | Y | Y | Y | Y | Y | 7/9 | Low |
| Morishita et al., 2021 | Y | N | U | Y | Y | Y | Y | Y | U | 6/9 | Moderate |
| Ohara et al., 2022 | U | N | U | Y | N | U | Y | N | U | 2/9 | High |
| Pérez-González et al., 2021 | U | U | Y | Y | U | Y | Y | Y | U | 5/9 | Moderate |
| Quandt et al., 2011 | Y | Y | Y | Y | Y | Y | Y | Y | Y | 9/9 | Low |
| Shiota et al., 2023 | Y | U | Y | Y | U | Y | Y | Y | U | 6/9 | Moderate |
| So et al., 2010 | Y | Y | Y | Y | U | Y | Y | Y | U | 7/9 | Low |
| Stankeviciene et al., 2021 | U | Y | Y | Y | U | Y | Y | Y | N | 6/9 | Moderate |
| Tanaka et al., 2023 | Y | Y | Y | Y | Y | Y | Y | Y | N | 8/9 | Low |
| Thomson et al., 1999 | U | U | Y | Y | Y | U | Y | Y | N | 5/9 | Moderate |
| Thomson et al., 2021 | Y | U | Y | Y | N | Y | Y | Y | U | 6/9 | Moderate |
| Wang et al., 2020 | Y | N | Y | Y | U | U | Y | Y | U | 5/9 | Moderate |

Y = Yes; N = No; U = Unclear; NA = Not Applicable

Q1: Was the sample frame appropriate to address the target population?

Q2: Were study participants sampled in an appropriate way?

Q3: Was the sample size adequate?

Q4: Were the study subjects and the setting described in detail?

Q5: Was the data analysis conducted with sufficient coverage of the identified sample?

Q6: Were valid methods used for the identification of the condition?

Q7: Was the condition measured in a standard, reliable way for all participants?

Q8: Was there appropriate statistical analysis?

Q9: Was the response rate adequate, and if not, was the low response rate managed appropriately?

Overall Risk of Bias Categorization: Low risk (7-9 "Yes" answers); Moderate risk (4-6 "Yes" answers); High risk (0-3 "Yes" answers).

Munn Z, Moola S, Lisy K, Riitano D, Tufanaru C. Chapter 5: Systematic reviews of prevalence and incidence. In: Aromataris E, Munn Z (Editors). JBI Manual for Evidence Synthesis. JBI, 2020
